# Why Are There So Few Basidiomycota and Basal Fungi as Endophytes? A Review

**DOI:** 10.3390/jof10010067

**Published:** 2024-01-15

**Authors:** Nattawut Rungjindamai, E. B. Gareth Jones

**Affiliations:** 1 Department of Biology, School of Science, King Mongkut’s Institute of Technology Ladkrabang (KMITL), Chalongkrung Road, Ladkrabang, Bangkok 10520, Thailand; 2 Department of Botany and Microbiology, College of Science, King Saud University, P.O. Box 2455, Riyadh 11451, Saudi Arabia; torperadgj@gmail.com

**Keywords:** basal fungi, basidiomycota, endophytes, fungal diversity

## Abstract

A review of selected studies on fungal endophytes confirms the paucity of Basidiomycota and basal fungi, with almost 90% attributed to Ascomycota. Reasons for the low number of Basidiomycota and basal fungi, including the *Chytridiomycota*, *Mucoromycota*, and *Mortierellomycota*, are advanced, including isolation procedure and media, incubation period and the slow growth of basidiomycetes, the identification of non-sporulating isolates, endophyte competition, and fungus–host interactions. We compare the detection of endophytes through culture-dependent methods and culture-independent methods, the role of fungi on senescence of the host plant, and next-generation studies.

## 1. Introduction

Studies of endophytic fungi have largely focused on bioprospecting for antimicrobials and the enhancement of plant growth, leaving a number of topics unexplored. Liu-Xu et al. [1] reviewed studies on endophytes by pooling data from papers published over the past 25 years, focusing on advances made, and highlighting topics that remain unresolved. They pointed out that most studies centered around ubiquitous Ascomycota, while Basidiomycota were poorly represented, sentiments also expressed by Adhikari et al. [2]. Yet, our studies of endophytes of the oil palm, *Elaeis guineensis*, in Thailand documented a number of basidiomycetes, initially identified by clamp connections in non-sporulating isolates, but when grown on selective media, produced micro fruitbodies with basidia and basidiospores [3,4]. In this review of selected papers on endophytes, we focus on why so few Basidiomycota and basal fungi have been found, whether they carry out bioactivities like those of Ascomycota, and whether they become saprophytes on senescence of the host plant, as suggested by Hyde and Soytong [5]? The papers selected were recently published and included data on endophytic Basidiomycota or basal lineages.

### 1.1. Diversity of Fungi

Fungi are a diverse group with an estimated 2.2–3.8 million species [6], with Ascomycota the most numerous (93,000 species) and Basidiomycota as the second largest phylum (40,000 species) [7,8]. Groups of endophytes can be defined according to various criteria, for example, host range, geographic distribution, the types of tissue that they colonize, modes of transmission, and benefits to host plants [9,10]. In terms of their taxonomy, endophytic fungi can be broadly classified into three main categories, namely, (1) Ascomycota, (2) Basidiomycota, and (3) basal fungi, including *Mucoromycota*, *Chytridiomycota*, and *Zygomycota* [11]. These studies [12,13,14] highlight that Ascomycota are the dominant group of endophytes and are worldwide in their distribution.

### 1.2. What Are Endophytes?

The term “endophyte” was firstly coined by De Bary in 1866 [15], who microscopically observed the presence of microbial cells in plant tissues and defined it as *“any organism that grows with plant tissues”*. Various definitions were proposed by later mycologists, but the one widely regarded is by Petrini [16] which described them as *“all organisms inhabiting plant organs that at some time in their life, can colonize internal plant tissues without causing apparent harm to their host”.* Research on endophytes has mainly focused on their ecology and bioprospecting for new metabolites, the latter because they are considered a treasure trove of bioactive compounds. Their bioactivity has been widely studied and reported, including antibacterial, antifungal, antiviral, anticancer, and immunosuppressive activities [17,18]. Research associated with microbial endophytes has dramatically increased over the last two decades with worldwide studies of diverse host plants and animals. Among endophytic microorganisms as a source of antimicrobials, fungi have been extensively studied and proven to be good candidates because they do not only produce a wide range of bioactive compounds but also possess plant-promoting factors and biological control activities against plant pathogens [19]. Bhunjun et al. [20] estimate that there are around one million fungal endophytes but believe this could well be nearer to three million. 

### 1.3. Mutualistic Nature of Plants and Endophytes

Symbiotic relationships between fungi and plants play an essential role in maintaining their good health, providing protection from abiotic and biotic stress, plant pathogens, and herbivores, and enhancing growth and yield [21,22]. Mycorrhizal fungi and dark septate endophytes (DSE) colonize the plant roots, and they play an essential role in plant growth and fitness [23]. The main difference between these two groups of fungi is their dependency on plants. Mycorrhizal fungi are strictly obligate symbionts, while endophytes can be either obligate or facultative plant symbionts [19,24]. Endophytes may complete their life cycle outside their host plants, so they are culturable on artificial media and found in all parts of plants both below and above ground [25]. 

### 1.4. Objectives of This Review

This review focuses on recent publications that reported basidiomycetes and basal fungi as endophytes, with a total of 24 publications selected from a broad range of studies of endophytes originally isolated from different plant species, various plant parts, and diverse geographical locations. In this article, we undertake a literature search of over a decade-long period (2008 to 2023) and examine the lifestyle of Basidiomycota and basal fungi as endophytes, because their occurrence remains equivocal and their ecological roles understudied [26]. We also discuss why their occurrence as endophytes is significantly lower when compared to their Ascomycota counterparts. The objectives of this review are as follows:

(1) To determine the percentage occurrence of Basidiomycota and basal fungi as endophytes as documented in selected published papers and to compare this with that of endophytic Ascomycota.
(2) To compare the diversity of fungi using culture-dependent (CD) and culture-independent (CID) methods based on a literature search.
(3) To discuss factors affecting the diversity and occurrence of endophytic Basidiomycota and basal fungi.
(4) To recommend procedures and methods to detect a wider range of endophytic Basidiomycota and basal fungi.

## 2. Diversity of Endophytes

### 2.1. Ascomycetous Endophytes 

From a review of 23 selected publications, Ascomycetous endophyte numbers are expressed as a percentage of the total number isolated and varied from 3.1 to 99.6% depending on the host plant and methodology used (Table 1). From all publications based on the isolation of axenic cultures of endophytes, there are three publications where percentage occurrences of Ascomycota were less than 50%, with 46%, 37%, and 23% from *Nothofagus pumilio* and *N. dombeyi* [27], *Pinus sylvestris* [28], and *Colobanthus quitensis* [29], respectively. For most publications (15 reports) based on isolation, the percentage occurrence was generally higher, between 52 and 99.6%. Ten reports had percentage occurrences of Ascomycota above 90% [3,30,31,32,33,34,35,36,37,38]. This finding is consistent with other reports on endophytes concluding that Ascomycota are the dominant taxonomic group encountered [13,20,39,40,41,42].

**Table 1:** Ascomycetous endophytes from 23 selected studies between 2008 and 2023.

Host	Plant Parts	No. of Ascomycetes	Total Number of Isolates	% Occurrence	Reference
*Colobanthus quitensis*	Leaves of angiosperm	6	26	23%	[29]
*Pterocladiella capillacea*	Red alga	2600	3187	81.58%	[43]
*Magnolia candolli* & *M. garrettii*	Leaves	54	56	96.5%	[30]
*Chloranthus japonicus*	Leaves, roots, and stem	317	325	97.5%	[31]
*Zostera marina*	Leaf of seagrass	103	110	93.6%	[32]
*Phragmites australis,* *Suaeda glauca* & *Limonium tetragonum*	Roots	153	156	98%	[33]
*Nothofagus pumilio* & *N. dombeyi*	Sapwood tissue	ND	210	46%	[27]
*Anacamptis morio*	Roots of orchids				[44]
*Myrtus communis*	Leaves of true myrtle	7 OTUs	44 OTUs	16%	[45]
*Stipa krylovii*	Roots	110	135	81.5%	[46]
*Nicotiana benthamiana,* *N. occidentalis* & *N. simulans*	Leaves, stems, and roots	ND	300	97.9%	[34]
63 Species of native plants	Stems and leaves	341	349	97.7%	[35]
*Sophora tonkinensis*	Phloem and xylem of roots of medicinal plant	36	47	76.6%	[47]
*Elaeis guineensis*	Leaves, petioles, rachis, and roots	ND	376	ND	[48]
*Vitis vinifera*	Leaves	239	240	99.6%	[49]
*Hevea brasiliensis* & *H. guianensis*	Sapwood and leaves of rubber tree	ND	2500	ND	[50]
*Nothapodytes nimmoniana*	Stem	44	45	98%	[36]
*Solanum cernuum*	Leaves and stems	33	55	60%	[38]
*Populus tremula*	Leaves of European aspen	93	96	97%	[37]
*Holcus lanatus*	Leaves and roots	337	348	97%	[51]
*Elaeis guineensis*	Petioles, rachides, vein, and intervein of leaves	320	340	94.1%	[3]
*Pinus sylvestris*	Sapwood tissue	53	143	37%	[28]
*Theobroma gileri*	Stem and pod tissues	16	31	52%	[52]

### 2.2. Basidiomycetous Endophytes

There are approximately 40,000 Basidiomycota taxa [53] exhibiting great diversity as yeasts, rust and smut fungi, endophytes, phytopathogens, and human pathogens, in terrestrial and aquatic habitats and from cold to temperate or tropical environments [53,54,55]. Table 2 documents endophytic Basidiomycota from a review of 25 selected publications from a wide range of host plants and plant parts, and from temperate to tropical geographical locations, with identifications based on morphology or a combination of morphology and sequence data, yielding 85 species in 46 genera. Two publications document the detection of endophytes in *Myrtus communis* and decaying leaves of *Halophila stipulacea* by high-throughput sequencing (metagenomics) (culture-independent method, CID) [40,45], while 23 publications identify the endophytes by isolation and morphological procedures (culture-dependent method, CD). 

Basidiomycetous endophytes numbers are expressed as a percentage of the total number isolated and varied from 0.4 to 84% depending on the host plant: leaves of *Vitis vinifera* yielded few basidiomycetous endophytes (0.4%) [49], while the highest number was from leaves of *Myrtus communis* (84%) [45]. Generally, basidiomycetous endophytes were low at 2.1%, in comparison to those of Ascomycota, in leaves, stems and roots of *Nicotiana benthamiana*, *N. occidentalis,* and *N. simulans* [56]. The number of basidiomycetous endophytes isolated per study vary greatly: 585 isolates from the red alga *Pterocladiella capillacea* [43], 310 isolated from sapwood and leaves of two rubber trees (*Hevea brasiliensis* and *H. guianensis*) [50], and with only one isolate of *Athelia* sp. from leaves of *Vitis vinifera*, and *Irpex lacteus* from the stem of *Nothapodytes nimmoniana* [36,49]. 

In general, the percentage occurrence of basidiomycetous endophytes was less than 5%. For example, two basidiomycetes (*Coprinellus magnolia* and *Phanerina mellea*) were reported as endophytes from a total of 56 isolates from leaves of *Magnolia candolli* and *M. garrettii* with a 3.5% occurrence [30]. Endophytes were isolated from leaves of *Elaeis guineensis*, and 10 of 376 isolates belonged to three species within Basidiomycota (*Neonothopanus nambi*, *Schizophyllum commune*, and *Ganoderma orbiforme*), accounting for a 2.7% occurrence [48]. A similar trend was reported by Marquez et al. [51], with 348 endophytes isolated from leaves and roots of *Holcus lanatus*, of which 11 isolates were basidiomycetous endophytes, with a 3% occurrence. These 11 isolates included *Agrocybe pediades*, *Coprinellus disseminates*, *Coprinus micaceus*, *Ceratobasidium* sp., *Cryptococcus podzolicus*, and *Rhodotorula slooffiae*. Many of these basidiomycetous endophytes are well-known wood-decaying fungi, highlighting their potential role in the senescence of the host tissue.

Generally, high-throughput sequencing yields a higher number of detected basidiomycetes. For this approach, DNA sequences are detected and reported as operational taxonomic units (OTUs). Abdel-Wahab et al. [40] also used metagenomics to determine the fungal diversity from five decaying leaf samples of *Halophila stipulacea* and found that the percentage occurrence of Basidiomycota ranged from 37.2 to 51.6%. Interestingly, the highest percentage of basidiomycetous endophytes was reported from leaves of *Myrtus communis* by Vas et al. [45]: out of 44 OTUs generated by sequencing, 37 OTUs belonged to 12 orders of Basidiomycota (84% occurrence).

**Table 2:** Basidiomycetous endophytes from 24 selected studies between 2008 and 2023.

Host	Plant Parts	Genus/Species	No. of Basidiomycetes	Total Number of Isolates	% Occurrence	Reference
*Colobanthus quitensis*	Leaves of angiosperm	*Lenzites* sp. *Leucosporidium* sp. *Peniophora* sp. *Phlebia* sp. *Sistotrema* sp. *Trametes* sp.	20	26	77%	[29]
*Halophila stipulacea*	Decaying leaves of seagrass	*Antrodiopsis* sp. *Malassezia* sp.	ND	296 OTUs	37.2–51.6%	[40]
*Pterocladiella capillacea*	Red alga	*Apiotrichum laibachii* *Bjerkandera adusta* *Cerrena* sp. *Chondrostereum* sp. *Grammothele fuligo* *Pseudozyma hubeiensis* *Rhodosporidium fluviale* *Rhodotorula mucilaginosa* *Tritirachium oryzae*	585	3187	18.36%	[43]
*Magnolia candolli* & *M. garrettii*	Leaves	*Coprinellus magnolia* *Phanerina mellea*	2	56	3.5%	[30]
*Chloranthus japonicus*	Leaves, roots, and stems	*Ceriporia* sp. *Thanatephorus* sp.	7	325	2%	[31]
*Zostera marina*	Leaf of seagrass	*Naganishia* sp. *Pseudozyma* sp. *Rhodotorula* sp.	4	110	3.6%	[32]
*Phragmites australis, Suaeda glauca* & *Limonium tetragonum*	Roots	*Meira* sp. *Pseudozyma* sp.	3	156	1.9%	[33]
*Nothofagus pumilio* & *N. dombeyi*	Sapwood tissue	*Armillaria sparrei* *Aurantiporus albidus* *Coprinellus* sp. *Fistulina antarctica*, *Hypholoma frowardii* *Laetiporus portentosus* *Obba valdiviana* *Pholiota baeosperma* *Postia pelliculosa* *Pseudoinonotus crustosus* *Sistotrema brinkmanni*	ND	210	43%	[27]
*Anacamptis morio*	Roots of orchids	*Ceratobasidium* sp. *Tulasnella* sp.	7	37	19%	[44]
*Myrtus communis*	Leaves of true myrtle	*Aurantiporus* sp. *Botryobasidium* sp. *Calocera* sp. *Ceratobasidium* sp. *Dacrymyces* sp. *Filobasidium* sp. *Flagelloscypha* sp. *Ganoderma* sp. *Gloeoporus* sp. *Gymnopilus* sp. *Hyphoderma* sp. *Hyphodontia* sp. *Hymenochaete* sp. Hymenochaetaceae sp. Hymenochaetales sp. *Malassezia* sp. *Naganishia* sp. *Phragmidium* sp. *Physisporinus* sp. Polyporaceae sp. Pterulaceae sp. *Pycnoporus* sp. *Rhodotorula* sp. *Sporobolomyces* sp. *Sympodiomycopsis* sp. Thelephorales sp. *Trametes* sp. Tricholomataceae sp. *Tyromyces* sp.	37	44	84%	[45]
*Stipa krylovii*	Roots	*Hymenochaete* sp. Tricholomataceae sp. Unknown fungi	25	135	18.5%	[46]
*Nicotiana benthamiana*, *N. occidentalis* & *N. simulans*	Leaves, stems, and roots	ND	ND	300	2.1%	[34]
63 Species of native plants	Stems and leaves	*Coprinopsis episcopalis* *Coprinus cinereus* *Cryptococcus* sp. *Filobasidium chernovii* *Ustilago* sp.	8	8	2.3%	[35]
*Sophora tonkinensis*	Phloem and xylem of roots of medicinal plant	*Fomitopsis* sp. *Exobasidiomycetidae* sp. *Schizophyllum commune* *Trichosporon asahii*	4	47	8.5%	[47]
*Elaeis guineensis*	Leaves, petioles, rachis, and roots	*Ganoderma orbiforme* *Neonothopanus nambi* *Schizophyllum commune*	10	376	2.7%	[48]
*Vitis vinifera*	Leaves	*Athelia* sp.	1	240	0.4%	[49]
*Hevea brasiliensis* & *H. guianensis*	Sapwood and leaves of rubber tree	*Bjerkandera* sp. *Ceriporia* sp. *Coprinellus* sp. *Peniophora* sp. *Phanerochaete* sp. *Phlebia* sp. *Rigidoporus* sp. *Stereum* sp. *Tinctoporellus* sp. *Trametes* sp.	310	2500	12.4%	[50]
*Nothapodytes nimmoniana*	Stem	*Irpex lacteus*	1	45	2%	[36]
*Solanum cernuum*	Leaves and stems	Basidiomycota sp. *Coprinellus radians* Coprinaceae sp. *Flavodon* sp. *Hohenbuehelia* sp. *Kwoniella mangroviensis* Meruliaceae sp. *Oudemansiella* sp. *Oudemansiella canarii* *Peniophora* sp. *Phanerochaete sordida* *Phanerochaete subserialis* *Phlebiopsis* sp. Polyporales sp. *Schizophyllum umbrinum*	21	55	38%	[38]
*Populus tremula*	Leaves of European aspen	Agaricomycetes sp. Sporidiobolaceae sp.	3	96	3%	[37]
*Holcus lanatus*	Leaves and roots	*Agrocybe pediades* *Ceratobasidium* sp. *Coprinellus disseminates* *Coprinus micaceus* *Cryptococcus podzolicus* *Rhodotorula slooffiae*	11	348	3%	[51]
*Elaeis guineensis*	Petioles, rachides, vein, and intervein of leaves	*Fomitopsis meliae* *Fomitopsis ostreiformis * *Fomitopsis pinicola* *Perenniporia* sp. *Pycnoporus sanguineus* *Schizophyllum commune* *Trametes lactinea*	20	340	5.9%	[3]
*Pinus sylvestris*	Sapwood tissue	*Bjerkandera adusta* *Heterobasidion annosum* *Peniophora* sp. *Schizophyllum commune* *Sistotrema coroniferum* *Thanatephorus cucumeris* *Trametes versicolor*	17	143	12%	[28]
*Theobroma gileri*	Stem and pod tissues	*Coprinellus* sp. *Ganoderma* sp. Lacnocladiaceae sp. *Lentinus* sp. *Melanotus* sp. *Meripilus* sp. *Piptoporus* sp. Polyporaceae sp. *Pycnoporus* sp. *Schizophyllum* sp. *Scopuloides* sp. *Wrightoporia* sp.	15	31	48%	[52]

Note: ND = No data.

### 2.3. Basal Fungi as Endophytes

In this review, 18 references documented basal fungi as endophytes (Table 3), of which 2 studies were performed by cultural-independent method (CID) [40,57], while the others were conducted by cultural-dependent methods (CD). Basal endophytic fungi belong to the phyla Mucoromycota and Mortierellomycota, recurring nine and six times, respectively. The Chytridiomycota are less frequent, with two reports, while other phyla including the Neocallimastigomycota, Entomophthoromycota and Kickxellomycota were recorded only once as endophytes.

The percentage occurrences of basal fungi as endophytes are generally lower than Basidiomycota and Ascomycota, ranging from 0.06 to 18% occurrence. The highest percentage is reported by Perkins et al. [58], with eleven endophytic isolates (18% occurrence) from kelp (*Ecklonia radiata*) and two isolates identified as *Mucor circinelloides.* The lowest percentage is reported by Cha, et al. [43] with 2 isolates of *Mucor irregularis* from 3187 endophyte isolates from the red alga *Pterocladiella capillacea*. In a metagenomic study of the decaying leaves of the seagrass *Halophila stipulacea* [40], six groups of basal fungi (and their percentage occurrence) belonging to Mucoromycota (12.56%), Chytridiomycota (5.4%), Mortierellomycota (11.58%), Neocallimastigomycota (13%), Entomophthoromycota (no data), and Kickxellomycota (no data) were documented. Abdel-Wahab, et al. [40] reported Chytridiomycota from a CD study: *Entophlyctis* and *Geranomyces* species with a 5.4% occurrence, while Kohout et al. [57] detected six OTUs as Chytridiomycota endophytes originally isolated from roots of submerged aquatic plants, with 3–10% occurrence. Mortierellomycota endophytes were identified in six studies with a single *Mortierella* species per study: roots of the orchid *Anacamptis morio* [44], and *Sophora tonkinensis* [47]; leaves of *Holcus lanatus* [51] and an unidentified isolate of the Mortierellales from roots of *Arabidopsis thaliana* [59]. When CID was used, Jin et al. [60] documented 12 sequences from *Stellera chamaejasme* (later identified as *Mortierella* spp.), accounting for 8% occurrence. Likewise, Abdel-Wahab et al. [40] also reported the occurrence of a *Mortierella* OUT (1.6%) when they studied fungal diversity of decaying leaves of *Halophila stipulacea*. 

For the Mucoromycota, four genera are recorded as endophytes including *Absidia*, *Mucor*, *Rhizopus*, and *Umbelopsis*. *Mucor* is the most recurring basal endophytic genus recorded from 11 publications in this review, all in very low numbers and as few isolates per study [38,43,47,58,60,61]. However, Jin et al. [62] reported 34 isolates of *Mucor* with a number of new species: *M. racemosus*, *M. hiemalis*, and *M. circinelloides* from *Stellera chamaejasme*, accounting for a 4.8% occurrence. Molina et al. [27] also reported a number of Mucoromycota from a study of the sapwood tissues of *Nothofagus pumilio* and *N. dombeyi.* A total of 88 endophytes were isolated on two culture media incubated at 20–24 °C for up to 4 months, yielding 10 isolates of *Umbelopsis* (*U. vinacea*, *U. changbaiensis*, *U. ramanniana*, and *U. nana/dimorpha*). Likewise, Abdel-Wahab et al. [40] also revealed the occurrence of Mucoromycota with a 12.6% occurrence when using metagenomics to survey decaying leaves of *Halophila stipulacea*. 

*Rhizopus* species have also been reported as endophytes: *R. oryzae* from *Opuntia ficus-indica* in Egypt [63]; *Rhizopus* sp. from the root of *Astragalus membranaceus* in China [64]; and two isolates of *Rhizopus* sp. from leaves of *Ziziphus spina* in Iraq [65]. The genus *Absidia* has been recorded twice as an endophyte: *Absidia* sp. from *Hedychium spicatum* [61] and *Absidia cylindrospora* from a root of *Arabidopsis thaliana* [59].

**Table 3:** Basal Fungi as endophytes and other fungi from 18 selected studies between 2008 and 2023.

Host	Plant Parts	Phylum	Genus/Species	No. of Isolates	Total Number of Isolates	% Occurrence	Reference
*Opuntia* *ficus-indica*	Cladodes of cactus	Mucoromycota	*Rhizopus oryzae*	1	ND	ND	[63]
*Ziziphus spina*	Leaves	Mucoromycota	*Mucor* sp. *Rhizopus* sp.	6	26	23%	[65]
*Halophila stipulacea*	Decaying leaves of seagrass	Mucoromycota	*Mucor* sp.	ND	ND	12.56%	[40]
		Chytridiomycota	*Entophlyctis* sp. *Geranomyces* sp.			5.42%	
		Mortierellomycota	*Mortierella* sp.			11.58%	
		Neocallimastigomycota	*Neocallimastix* sp. *Anaeromyces* sp.			13.31	
		Entomophthoromycota	Unknown fungi			ND	
		Kickxellomycota	Unknown fungi			ND	
*Ecklonia radiata*	Kelp	Mucoromycota	*Mucor circinelloides*	2	11	18%	[58]
*Pterocladiella capillacea*	Leave of red alga	Mucoromycota	*Mucor irregularis*	2	3187	0.06%	[43]
*Zostera marina*	Leave of seagrass	Mucoromycota	*Absidia cylindrospora*	1	120	0.9%	[32]
*Nothofagus pumilio* & *N. dombeyi*	Sapwood	Mucoromycota	*Umbelopsis vinacea* *U. changbaiensis* *U. ramanniana* *U. nana/dimorpha*	10	88	11%	[27]
*Astragalus membranaceus*	Roots	Mucoromycota	*Rhizopus* sp.	1	ND	-	[64]
*Anacamptis morio*	Roots	Mortierellomycota	*Mortierella* sp.	1	37	3%	[44]
*Hedychium spicatum*	Rhizome and leaves	Mucoromycota	*Absidia* sp. *Mucor hiemalis*	2	28	7%	[61]
*Sophora tonkinensis*	Phloem and xylem of roots of a medicinal plant	Mortierellomycota	*Mortierella alpina*	1	42	2%	[47]
		Mucoromycota	*Mucor circinelloides*	1		2%	
*Stellera chamaejasme*	Leaves, stems, and roots of a medicinal plant	Mortierellomycota	*Mortierella* spp.	12	145	8%	[60]
		Mucoromycota	*Mucor* sp. *Rhizopus* sp.	2		1.4	
*Arabidopsis thaliana* & *Microthlaspi perfoliatum*	Roots	Mucoromycota	*Absidia cylindrospora*	1	100	1%	[59]
		Mortierellomycota	Mortierellales sp.	1		1%	
*Stellera chamaejasme*	Leaves, stems, and roots of a medicinal plant	Mucoromycota	*Mucor racemosus* *M. hiemalis* *M. circinelloides*	34	714	4.8%	[62]
*Isoetes echinospora* *Isoetes lacustris* *Littorella uniflora* *Lobelia dortmanna* *Subularia aquatica*	Roots of submerged aquatic plants	Mucoromycota	OUT28	2	234 OTUs	0.08%	[57]
		Chytridiomycota	OTU34, 35 & 36 OTU29, 30 & 27	6		2.6%	
*Solanum cernuum*	Leaves and stems	Mucoromycota	*Mucor* sp.	1	55	1.8%	[38]
*Pinus sylvestris*	Sapwood tissue	Mucoromycota	*Mucor hiemalis* *Mucor plumbeus* *Rhizopus stolonifer* *Umbelopsis isabellina* *Umbelopsis vinacea*	8	143	6%	[28]
		Mortierellomycota	*Mortierella globalpina* *Mortierella lignicola*				
*Holcus lanatus*	Leaves	Mortierellomycota	*Mortierella* sp.	1	214	0.5%	[51]

### 2.4. Frequency of Endophytic Species of Basidiomycota and Basal Fungi 

Species frequency of Basidiomycota and basal fungi as endophytes isolated from culture-dependent (CD) and culture-independent (CID) methods are compared (Table S1 in the supplement data). The frequency of Basidiomycota is far greater than basal fungi in both methods and its frequency accounts for more than three quarter of the species. When data on CD and CID were combined, order frequency was studied. Within the twenty-one orders of Basidiomycota taxa contributing as endophytes, the Polyporales and Agaricales were the most frequently cited in the literature. Both appear 16 times from 25 selected references. Four other orders include *Cantharellales*, *Tremellales*, *Russulales*, and *Sporidiobolales* and were reported eight, six, five, and five times from the same set of publications, while a further fifteen orders were reported only once. Seven orders of basal fungi were represented as endophytes, with the *Mucorales* appearing 13 times from 17 selected publications. The *Mortierellales* is the second most frequent, appearing six times, while the other five orders including the *Chytridiales*, *Endogonales*, *Monoblepharidales*, *Rhizophydiales*, and *Umbelopsidiales* are less frequent and appear only once. 

#### 2.4.1. Basidiomycota

Table S1 (the Supplementary Data) lists the frequency of endophytic Basidiomycota and basal fungi reported from the selected 25 publications in our survey, with 66 species in 92 genera. *Schizophyllum commune* was the most recurring species, occurring on four host plants, while the genera *Ceratobasidium* and *Trametes* were listed three times in various host plants. *Ceratobasidium* species were recorded from the root of *Anacamptis morio* [44], the leaf of *Myrtus communis* [45], and the grass *Holcus lanatus* [51], with *Trametes* species reported from *M. communis* [45], *Hevea brasilienesis* [50], and *Colobanthus quitensis* [29]. Seven Basidiomycota genera were listed twice as endophytes: *Bjerkandera adusta* [28,43], *Ganoderma* sp. [45,52], *Naganishia* sp. [32,45], *Rhodotorula* sp. [32,45], *Hymenochaete* sp. [45,46], *Coprinellus* sp. [27,50], and *Phlebia* sp. [29,50]. Other basidiomycetous endophytes were reported only once.

Most endophytic basidiomycetous are filamentous, but eight were yeasts, accounting for a 17.4% occurrence. Most of them are cosmopolitan and distributed worldwide. The most frequent basidiomycetous yeast genus was *Rhodotorula* (occurring four times in different host plants and locations): *Rhodotorula* sp. on a leaf of *Myrtus communis* [45] and *Zostera marina* [32]; *R. slooffiae* on a leaf of *Holcus lanatus*, [51] and *R. mucilaginosa* found on the red alga (*Pterocladiella capillacea*). Four basidiomycetous yeasts were less frequent and found twice, namely, *Cryptococcus* [35,51], *Filobasidium* [35,45], *Malassezia* [40,45], and *Pseudozyma* [33,43]. A further three yeast genera were found once as endophytes: *Rhodosporidium* [43], *Sporobolomyces* [45], and *Trichosporon* [47]. Some yeasts are common microflora on human skin, particularly *Malassezia*. However, culture-independent studies of fungi from environmental samples showed that *Malassezia* are exceedingly widespread and ecologically diverse, from polar regions to deep-sea vents [66].

#### 2.4.2. The Basal Fungi

There are nine genera and 13 species of basal fungi reported as endophytes, with the genus *Mucor* being the most frequently listed in eight publications (Table S1). Five endophytic *Mucor* species include *Mucor* sp. [38,40], *M. circinelloides* [47,58,62], *M. irregularis* [43], *M. hiemalis* [61,62], and *M. racemosus* [62]. Nineteen and three species were recorded by CD and CID methods, with three genera recorded by both methods, including *Mucor*, *Mortierella*, and *Rhizopus*. 

## 3. Comparison of Culture-Dependent (CD) and Culture-Independent (CID) Methods

The diversity of fungi has traditionally been studied based on culture-dependent methods (CD) which rely on isolation and identification using morphological and molecular data. With advances in molecular methodology, culture-independent methods (CID), such as TGGE (thermal gradient gel electrophoresis), DGGE (denaturing gradient gel electrophoresis), SSCP (single-strand conformation polymorphism), RFLP (restriction fragment length polymorphism), TRFLP (terminal restriction fragment length polymorphism), ARDRA (amplified ribosomal DNA restriction analysis), pyrosequencing, and Illumina MiSeq sequencing, focus on extracting DNA directly from environmental samples without isolation of axenic cultures and are increasingly playing an important role in the discovery of hidden fungal species [67]. Five publications on fungal diversity used both CD and CID methods on the same specimens (Table 4), three focusing on endophytic fungi [14,68,69,70] and two fungi from decaying leaves of *Halophila stipulacea* and marine sediments for comparison [71]. 

**Table 4:** Direct comparison between culture-dependent and culture-independent methods from 5 publications.

Source	Culture-Dependent Method					Culture-Independent Method				Reference
	No. of Isolates	Phyla	Name	No. of Genera (Isolate)	% Occurrence	No. of OTUs	Phyla	Name	% of Occurrence	
Endophyte of aquatic plants	1689	3	Ascomycota	123 (1584)	93.8%	1074	6	Ascomycota	43.48%	[68]
			Basidiomycota	29 (92)	5.4%			Basidiomycota	15.36%	
			Zygomycota	2 (13)	0.8%			Zygomycota	1.49%	
								Chytridiomycota	1.21%	
								Glomeromycota	0.02%	
								Rozellomycota	0.01%	
								Unknown fungi	38.17%	
Endophytes of *Elymus repens*	66	1	Ascomycota	9 (27)	40.9%	48	4	Ascomycota	90%	[69]
			Unidentified fungi	Unknown (39)	59.1%			Basidiomycota	2%	
								Glomeromycota	2%	
								Mortierellomycota	2%	
								Unknown fungi	4%	
Endophytes of *Vitis vinifera*	94	1	Ascomycota	19 (94)	100%	59	3	Ascomycota	93.6%	[70]
								Basidiomycota	4.2%	
								Zygomycota	2.1%	
Endophytes of *Acanthus ilicifolius*	203	2	Ascomycota	30 (200)	97.04%	111	2	Ascomycota	65.09%	[14]
			Basidiomycota	2 (3)	2.96%			Basidiomycota	38.87%	
								Unknown fungal taxa	4.05%	
Deep-sea sediment	19	2	Ascomycota	11 (14)	73.7%	42	2	Ascomycota	59.5%	[71]
			Basidiomycota	2 (5)	26.3			Basidiomycota	40.5%	

Based on CD methods, the number of fungal isolates vary from 19 to 1689 isolates, with Ascomycota being dominant, accounting for between 40.9 [69] and100% occurrence [70]. This further confirms Ascomycota as the dominant group of endophytes. Three publications include data on Basidiomycota, accounting for 2.96, 5.4, and 26.3% occurrence [14,68,71], while none were reported in a study by Dissanayake et al. [70]. Zheng et al. [68] reported endophytic Zygomycota with 0.8% occurrence.

When the same set of source specimens was used for a CID study, Ascomycota were still predominantly common in all publications, but the percentage occurrence of Basidiomycota was greater than for the CD method. Zheng et al. [68] showed that the percentage occurrence of basidiomycetous endophytes from aquatic plants, isolated by CD methods, was 5.4%, but increased to 15.53% with CID methods. Likewise, Chi et al. [14] compared the occurrence of endophytes of mangrove leaves (*Acanthus ilicifolius*), and found that the occurrence of basidiomycetous endophytes was low at 2.96% (CD) but was significantly higher at 38.87% with CID methods. This was also reported for Basidiomycota from marine deep-sea sediments, with percentage occurrence increasing from 26.3 to 40.5% for CD and CID methods, respectively [71]. 

It is worth noting that using CID methods improves the chance of revealing other basal fungi which might be hidden in host substrates. This was confirmed by Zheng et al. [68], who found *Zygomycota* isolates present as endophytes (0.8% occurrence), but at least four basal phyla were detected as endophytes with a CID study, namely, *Zygomycota*, *Chytridiomycota*, *Glomeromycota*, and *Rozellomycota*, as well as a lineage of unknown fungi.

## 4. Factors Affecting the Occurrence of Basidiomycota and Basal Fungal Endophytes

This review has shown that the enumeration of endophytes depends on the methods employed, with the use of metagenomics resulting in a wider range of taxa, especially for Basidiomycota and basal fungi, However, many other factors affect successful detection of endophytes, including isolation procedure and media, sporulation, and identification of non-sporulating isolates. With Ascomycota widely reported as ubiquitous endophytes and significantly higher than other taxonomic groups, it raises the question as to why there are so few endophytic Basidiomycota and basal fungi found? Despite being abundant in nature and thriving in a wide range of substrata and environments, only a handful of Basidiomycota and basal fungi have been reported as endophytes [1,2]. Several factors may account for this and are considered here.

### 4.1. Isolation Procedure

It is universally acknowledged that there are no standard procedures for the surface sterilization of material when isolating endophytes [72]. Different sterilants, their concentration, and duration of application vary greatly from study to study. Successful methods for endophyte isolation have been reviewed [67,73]. Yu et al. [74] studied the effect of different concentrations of sodium hypochlorite employed and exposure times on the efficacy of surface sterilization on endophyte diversity from tea plants (*Camellia sinensis*). They found that stem and leaf tissues need different conditions, with mature stem tissue requiring a higher concentration and longer exposure time to achieve complete surface sterilization. Selecting an inappropriate surface sterilant may result in two outcomes: if too mild and the duration is too short, it may not eliminate phylloplane contaminants or epifoliar/epiphytic fungi on the surfaces of the host surface. If the sterilant is too strong and applied for too long, it may destroy endophytes and consequently generate ambiguous results [75]. Additionally, some studies suggested dissecting plant tissues into small pieces before performing surface sterilization; this might lead to lower isolation frequency because the sterilant might penetrate the inner tissue and kill endophytes. It is critically important to choose an appropriate sterilant, optimal concentration, and least exposure time because this ensures the elimination of contaminants and epiphytes without deterring endophytes. After choosing the plant of interest, a surface sterilization method should be selected from the relevant literature, and a preliminary study on surface sterilization including testing the efficacy of surface sterilization, e.g., by imprinting tissues onto agar and assessing for fungal growth, is recommended. 

### 4.2. Isolation Media

The composition of culture media may favor the growth of certain groups of fungi, while others may be suppressed. Potato dextrose agar (PDA) is the universally used isolation medium as it supports a wide range of fungi including yeasts and molds [76]. If the study is solely focused on a certain group of easily cultivable fungal endophytes, PDA is recommended as a single culture medium [77]. Other growth media used are malt extract agar (MEA), Czapek medium (Cz), tryptone soybean agar (TSA) [67], glucose–yeast extract-peptone seawater agar (GYPS), potato dextrose seawater agar (PDAS), and artificial seawater agar (SA) [43]. However, when studying the diversity of fungal endophytes, at least two or more mycological media should be employed in addition to PDA. To increase the chance of recovering more fungal taxa from host plants, Pinruan et al. [3] isolated endophytes from the oil palm *Elaeis guineensis* using PDA with added streptomycin sulphate (0.5 g dissolved in 1.5 mL sterile water per liter of agar), resulting in 1890 endophyte isolates from two samplings being classified into 340 morphotypes (taxa). Although most of the endophytes were ascomycetes, especially Xylariaceous species, twenty taxa belonged to Basidiomycota and were later identified at the molecular level using ribosomal DNA, LSU, and ITS sequence data (*Fomitopsis* cf. *meliae*, *F.* cf. *ostreiformis*, *F.* cf. *pinicola*, *Perenniporia* sp., *Pycnoporus sanguineus*, *Trametes lactinea*, and *Schizophyllum commune*). Many formed micro fruitbodies in culture and others were detected by the formation of clamp connections. 

Media with reduced water activity that are selected for osmotolerant, halotolerant, and xerotolerant fungi are also alternatives to be used as isolation media. When water potential is decreased, the growth rate of wood-rooting basidiomycetes is also decreased. But some isolates were able to grow at the lowest level, at −4.4 MPa [78]. Lu et al. [79] also isolated endophytes from *Cotoneaster multiflorus* using PDA. These endophytes were screened for drought tolerance on PDA amended with different concentrations of polyethylene glycol to stimulate osmotic potential; some isolates showed drought tolerance at the lowest level, −0.6 MPa. This procedure may encourage some endophytes hidden in plant tissue to grow on these types of media.

Molina et al. [27] isolated endophytes from sapwood tissue of *Nothofagus pumilio* and *N. dombeyi* using two culture media: (1) 2% dextrose corn meal agar medium amended with a 1% neomycin–penicillin–streptomycin solution and (2) Basidiomycota selective medium (1.5% malt extract agar with 40 mg benomyl, 20 mg dichloran, and 100 mg streptomycin sulphate per liter). They found a higher abundance of Basidiomycota with these media. Out of 210 isolates, 43% belonged to Basidiomycota. Benomyl and dichloran are fungicides which have inhibitory activity against most ascomycetous fungi, but they cause slight or no inhibition of basidiomycetous and zygomycotan fungi [80,81]. This confirms that in addition to PDA, other culture media should be used to enable the recovery of a wider range of taxa, in particular, the use of dichloran and benomyl in order to detect endophytic basidiomycetes. Hoff et al. [82] also employed selective media for the isolation of endophytes from Ponderosa pine (*Pinus ponderosa*) and Douglas-fir (*Pseudotsuga menziesii*). Although a medium selective for basidiomycetous fungi was used, the only basidiomycetes isolated were *Tremella* sp. (two isolations) and *Heterobasidion* sp. (one isolation). Most of the fungi isolated were ascomycetes and zygomycetes (Table 1). So clearly, basidiomycetes were rare in this study despite the use of specialized media. What is vital is that the incubation period for the isolation of Basidiomycota needs to be much longer than for other fungi [83,84,85].

Few studies have detected Chytridiomycota from intact plant tissues using metagenomics [57,86]. The majority of endophytic chytrids and other basal fungi were reported from roots of submerged aquatic plants [57]. However, Chytridiomycota have not been detected using culture-dependent methods. It is not surprising that Chytridiomycota are rarely encountered as endophytes as they are predominantly found in aquatic habitats and therefore may not find suitable host plants. Generally, baiting with sterilized seeds, leaves, pollen, or animal skin is the method use for their detection. Geisen et al. [87] suggested that a baiting technique should be combined with a surface sterilization method; after the host plant is surface sterilized, it is transferred to a Petri dish with sterile demineralized water with added sterile grass leaves (*Agrostis capillaris*) as baits. The dishes are then incubated at room temperature for 12 h to allow zoospores of chytrids within plant tissue to migrate and colonize the baits. Infected grass leaves are transferred to water agar (1.6% agar) containing streptomycin and subsequently to potato dextrose agar. This procedure offers an alternative method of recovering endophytic Chytridiomycota. 

### 4.3. Period of Incubation 

Although PDA offers rich nutrients for fungal growth, it may favor fast-growing fungi, especially ascomycetes, which might outperform and overgrow others. Meanwhile, basidiomycetes, which are generally slow growing, might not be able to compete with ascomycetes, resulting in lower numbers as endophytes. The incubation period may also greatly affect the number of endophytes. Lower temperatures and longer incubation periods may facilitate species recovery by reducing the growth of fast-growing mesophilic fungi as well as reducing the drying out of culture media. Hagh Doust et al. [88] isolated endophytes using two incubation temperatures (4 °C and 25 °C) and they found that using low temperature (4 °C) can increase the numbers of fungal endophytes isolated. This allowed psychrophilic and psychrotolerant fungi to be discovered. The period of incubation can range from 3 to 20 days and it can be extended for up to six weeks [67]. If the incubation is extended for weeks or months, it is necessary to seal the plates with Parafilm to maintain the humidity of media and minimize the risks of contamination. It usually takes between a few days to a few weeks for fungi to emerge from plant materials; therefore, the plates seeded with plant materials should be monitored daily. If hyphal growth is observed, it should be transferred onto a new plate immediately. Although there is no certain period which can guarantee the best yield of endophytes, the incubation period should be as long as possible to ensure that all endophytes including ascomycetes, basidiomycetes, and other basal fungi have equal chance to grow out from the host plant. Baum et al. [89] isolated fungi from wood immediately after felling, as well as after incubation for 8, 16, and 24 weeks. Only a few isolates were obtained from freshly cut wood, but a large number of isolates was recovered after eight weeks of wood incubation under sterile conditions. Basidiomycetes required an incubation period longer than ascomycetes to emerge from the tissue samples.

Cha et al. [43] isolated endophytes from a red alga, resulting in 585 isolates of endophytic basidiomycetous from a total of 3187 isolates, incubated and observed periodically for one month. Bertini et al. [29] successfully isolated 20 isolates (77% occurrence) of endophytic basidiomycetous from the Antarctic plant, *Colobanthus quitensis*, incubated for 60 days and assessed daily. This emphasizes the importance of extending the incubation period as long as possible to allow basidiomycetes and basal fungi to grow onto solid agar. 

### 4.4. Endophytic Yeasts—The Forgotten Bioresource

When mycologists study endophytes, they mostly focus on filamentous fungi on appropriate media. However, a serial dilution method is preferred for the isolation of yeasts from natural sources because it offers a better chance of yeast isolation and allows for a better recovery with less interference from mycelial fungal growth forming colonies on agar [90,91]. Two procedures are recommended: (1) plant material is cut into small fragments after surface sterilization, homogenized, serially diluted with normal saline solution, and spread plate onto solid agar [92], and (2) plant material is surface sterilized, cut into small fragments, inoculated into sterile broth aseptically to enable the growth of yeasts, and finally, serially diluted and spread on solid agar [90]. 

These two techniques are suitable for unicellular microbes and should be performed separately and independently from the isolation of filamentous endophytes. This would improve the chances of detecting endophytic yeasts from plant samples. Isaeva et al. [93] pointed out that plant inner tissue contains a high content of sugars and starch and may harbor a wide range of ascomycetous and basidiomycetous yeast genera. 

### 4.5. Identification 

Studies of endophytic fungi often result in sterile or non-sporulating cultures. In a study of fungal endophytes isolated from healthy leaves, rachises, and petioles of the oil palm *Elaeis guineensis* in a Thai plantation, 892 and 917 endophytes were isolated, yielding 162 and 178 morphotypes, respectively, with non-sporulating isolates grouping into 162 morphotypes according to their colony morphology [4]. Sporulating endophytes can be identified based on their spore characteristics and other unique features that they produce in culture media. 

The lowest proportion of sterile mycelia was 11–16% from *Trachycarpus fortunei* [94], with the highest proportion of 54% from *Quercus ilex* [95]. Gnavi et al. [96] isolated 88 endophytes from *Posidonia oceanica*, of which 21 (23%) were sterile mycelia, while Mattoo and Nonzom [97] isolated 681 endophytes from *Ephedra gerardiana*, but most (499 isolates, 73.2%) were sterile mycelia. 

However, non-sporulating endophytes are difficult to deal with because they only produce mycelium, without producing any other useful identifying characteristics. Various methods can be used to induce sporulation of endophytes. For example, Tanney and Seifert [98] induced sporulation using a variety of methods including (1) prolonged incubation at low temperatures (5 °C) and (2) floating mycelia blocks in sterile water. Additionally, Ibrahim et al. [99] identified a new species of *Xylaria* by connecting it with stromata occurring in nature, similar to the method described by Truong et al. [100]. 

The molecular identification of unknown endophytes is conducted by extracting genomic DNA from sterile mycelia and then followed by amplifying and sequencing internal transcribed spacers (ITS region), a DNA barcode for Kingdom Fungi. Then, the ITS sequence of an unknown sample is compared to various sequences deposited in the DNA databases. If an unknown sample is matched to a known sequence from published and reliable sources, this leads to successful identification. However, this method does not always lead to identification. On many occasions, unknown samples cannot be matched with sequences in databases.

**Figure 1:**
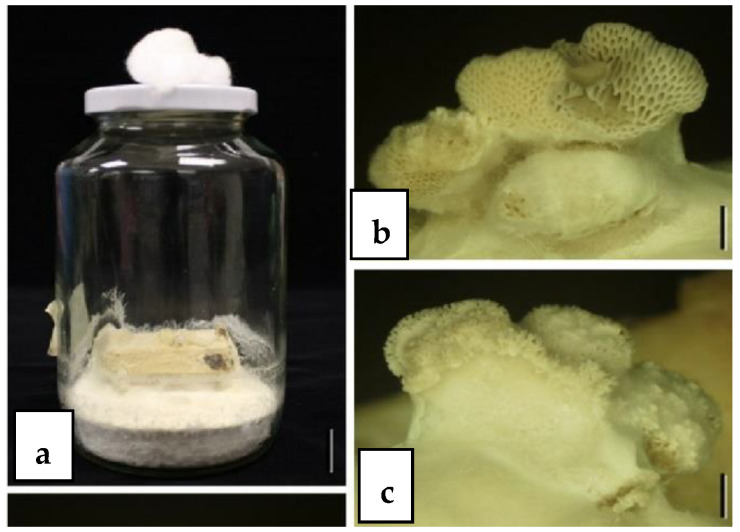
(**a**) Spore induction of *Fomitopsis* cf. *meliae*, a basidiomycetous endophyte from oil palm on a test block of oil palm petiole in a jar containing PDA medium. (**b**,**c**) Poroid fruiting bodies of *Fomitopsis* cf. *meliae* [4].

**Figure 2:**
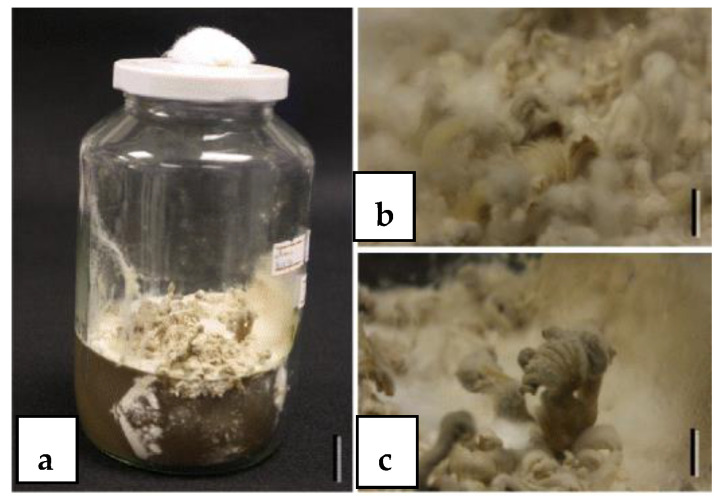
(**a**) Poroid and minute fruiting bodies produced in a jar containing PDA medium. (**b**,**c**) Fruiting body induction of *Schizophyllum commune* (basidiomycetous endophyte from oil palm) [3].

When endophytes fail to sporulate and identification is unsuccessful, attempts can be made to induce sporulation on culture media. Rungjindamai et al. [4] inoculated sterile endophytic isolates from *Elaeis guineensis* onto a test block of palm petiole on PDA. After 12 months of incubation, poroid and minute fruiting bodies were observed (Figure 1) and identified as *Fomitopsis meliae* by combined ITS sequence analysis. Pinruan et al. [3] used a similar technique to induce sporulation of sterile mycelia from oil palm by growing cultures in “jam” jars containing PDA and incubated for six days, resulting in basidiomes with a white cap and gills bearing basidia and basidiospores (Figure 2). Subsequently it was identified by ITS sequence analysis as *Schizophyllum commune*. 

These studies confirm that sterile mycelia can be induced to sporulate. However, this approach may need numerous attempts of trial and error. It is also laborious and time consuming with no guarantee of success because sporulation of fungi relies on various factors, for example, nutrition, host tissue, light, temperature, and humidity [101]. Therefore, molecular identification is needed. DNA sequence analysis using internal transcribed spacer (ITS region) sequencing is widely used for fungal identification and it has been proven to successfully circumvent the backlog of sterile mycelia [72]. This method leads to a rapid expansion of successful identification of sterile mycelia of endophytes. Screening non-sporulating endophytes for bioactive compounds can be used for their identification, especially xylariaceous fungi, for example, xylaranic acid [102], terpenoids [103,104,105,106,107], xanthones [108,109], cytochalasins [110], cyclopeptides [111], polyketides [112,113], and xyloketals [114].

### 4.6. Fungus–Host Interaction

Two issues can be considered here: endophyte entry into the host plant and fungal interactions within the host plant. Endophytes have been well studied, but how they gain entry into their hosts is far from clear and open to debate [20]. Vertical and horizontal transmission are generally cited, and while the former is well supported by studies [115], the latter may be through roots, stomata, or open wounds caused by human activities, insects, herbivores, and other predators. Host plants are known to be sensitive to attack by fungi and have defense mechanisms [116]. It is suggested that the presence of endophytes within the host plant suppresses incoming pathogens by the production of extracellular enzymes or secondary metabolites or toxins. Antifungal compounds include acetonic extracts of acetic acid and palmitic acid, jasmonic acid (JA), salicylic acid (SA), peroxidase (POD), polyphenol oxidase (PPO), rhinomilisin B, divirensol H, and trivirensol [117,118]. Duckett et al. [119] isolated basidiomycete endophytes from a jungermannialean (leafy) liverwort, confirming their presence by transmission electron microscopy due to characteristic dolipore septa. They also proposed that the fungi entered their hosts via the tips of the rhizoids and develop distinctive distributions within the liverwort “stem”.

Secondly, there are little experimental data on the interactions of endophytic fungi within host cells. Although there is no direct report on the interaction between Ascomycota and Basidiomycota as endophytes within host cells, Xie et al. [120] examined the interaction between *Alternaria* sp. and *Diversispora epigaea* (an arbuscular mycorrhiza, a basidiomycete) and their effect on the growth of maize. Both co-colonized maize roots improved the growth above and below ground by increasing plant growth and altering root morphology, respectively. 

## 5. General Discussion

### 5.1. Basidiomycota as Hidden Endophytes

From this review, it is clear that Ascomycota dominate endophytic diversity within host plants, although recent metagenomic studies suggest that basidiomycetes may be more common than previously anticipated. Conventional methods by isolation on PDA yields a lower number of Basidiomycota, but inclusion of benomyl and dichloran to the medium enhances the number of basidiomycetes isolated [48]. Thus, for the detection of a wider fungal diversity, at least two sets of media are required: (1) a general propose medium, for example PDA, MEA, and CMA for generalist endophytes, and (2) a selective medium, for example PDA added with benomyl and dichloran for basidiomycetous endophytes. This approach should increase chances of discovering hidden basidiomycetous endophytes which are already present in plant tissue. Endophyte studies using a culture-independent method (CID) showed that numerous basidiomycetous genera were detected and a higher percentage occurrence of Basidiomycota was recorded [14,40,68]. 

### 5.2. The Low Occurrence of Chytridiomycota as Endophytes

The Chytridiomycota are well studied as parasites and saprophytes in aquatic ecosystems in both freshwater and marine habitats [121,122,123,124,125,126], and are shown to be abundant in nature, forming a major component in food webs associated with zooplankton and phytoplankton [121]. However, few are reported as endophytes, mostly with a low 1% occurrence or less [86,127]. Abdel-Wahab et al. [40] reported a high percentage occurrence of Chytridiomycota (5.42%) fungi detected from decaying leaves of *Halophila stipulacea*, and this is in agreement with other published studies of this group, suggesting they are underpopulated in databases [128,129,130,131]. HTS studies report that the major lineages of fungi globally comprise 43% Ascomycota, 36% Chytridiomycota, and 27% Basidiomycota [132]. However, there is doubt as to whether chytrids are true endophytes when they are detected in leaves. The only way to solve this is to isolate chytrids from fresh and symptomless plant materials. 

Although endophytes are isolated from aquatic plants, Chytridiomycota are not detected in all plant samples collected from the aquatic ecosystem, with none reported from four seagrasses *Cymodocea serrulata*, *Enhalus acoroides*, *Halophila ovalis*, and *Thalassia hemprichii* in Thailand [133]; four species of freshwater plants *Persicaria amphibia*, *Stuckenia pectinate*, *Elodea bifoliata*, and *Myriophyllum sibiricum* in the US [134]; and five species of aquatic/riparian plants *Ottelia acuminata*, *Myriophyllum verticillatum*, *Equisetum arvense*, *Cardamine multijuga*, and *Impatiens chinensis* in China [135]. These examples confirm the paucity of endophytic chytrids highlighted by this review. This might be due to their nature as aquatic microbes. They have a short life cycle and spend time in their life form as motile zoospores, which might exclude an endophytic existence. Chytrids may also be unable to colonize and penetrate into host plants due to host–parasite specificity. Most of the plants surveyed for endophytes are terrestrial and may not come into contact with chytrids.

### 5.3. Other Basal Fungi as Endophytes

#### 5.3.1. The Mucoromycota

Endophytic Mucoromycota are poorly represented as endophytes, but they are found in diverse habitats from aquatic to terrestrial locations. *Mucor*, *Rhizopus*, and *Umbelopsis* are three common genera reported as endophytes [27,62], are cosmopolitan and widely dispersed by air, and are present in soils and decaying organic matter [136]. An endophytic fungus *Mucor* sp. CBRF59 was isolated from a healthy root of rape (*Brassica napus*) growing in heavy metal-contaminated soil [137]. Their paucity as endophytes may well be due to their inability to colonize host plants. 

#### 5.3.2. Mortierellomycota

Mortierellomycota occur in diverse habitats, form abundant filamentous growth in soil, and are worldwide in distribution [138,139]. Numerous genera of Mortierellomycota, such as *Mortierella*, are widely reported as root endophytes [57,140,141]. Is their poor documentation as endophytes due to poor identification? Mortierellomycota can grow on culture media and conditions commonly used for Ascomycota and Basidiomycota, but failure in their identification may be due to their lack of sporulation. As with other basal fungi, culture-independent studies have detected greater species diversity [40,69,142,143,144]. 

### 5.4. Role of Endophytes in the Senescence of Host Plants

Boddy and Griffith [145] investigated endophytes of young twigs of various timbers and found basidiomycetes *Peniophora lycii* in ash, and *Peniophora quercina* and *Vuilleminia comedens* on oak, but generally, most of the fungi were cosmopolitan asexual morphs. They emphasized that it is difficult to pin-point the exact time of death of host plants. Therefore, it is difficult to determine which fungi begin to colonize and when they started to decompose plants. They suggested that early colonization of plants occurs by a latent invasion by endophytes in living plant tissue. However, some ascomycetous genera, for example *Fusarium*, *Phomopsis*, and *Xylaria*, are commonly found as endophytes but are also frequently isolated from dead plants. Boddy and Griffith [145] concluded that several of the common endophytes of the sapwood and bark of deciduous trees are primarily saprotrophic, being specifically adapted to colonize and utilize dying host tissue. This emphasizes the blurring line between endophytes and saprophytes. Hyde and Soytong [5] also suggested that some endophytes become saprophytes after the senescence and death of the host plants, and this has acquired a degree of support from other studies [146,147,148]. Some endophytes become latent pathogens, weak parasites, and pathogenic and may later cause disease if the host plants are under stress [149]. Wenndt et al. [150] studied the decomposition process by endophytes of *Stipagrostis sabulicola* when these isolates were reinoculated into the tiller litter of the plant. Of the 20 endophyte taxa tested, 80% (16 taxa) became saprophytes by decomposing the litter over a 28-day assay, but 4 taxa were unable to decompose the litter. Their result confirms the hypothesis that not all endophytes become saprophytes after host death. Most studies have documented endophytic Ascomycota becoming saprophytes, with little reference to the role of endophytic Basidiomycota. Schwarze et al. [151] and Baum et al. [89] have proposed that fungal endophytes may initiate wood decay, although the exact mechanism is not understood. An endophytic basidiomycete from *Sphagnum fuscum* caused a 10.2% mass loss in spruce wood chips after 8 weeks [152]. Oses et al. [153] evaluated the role of basidiomycete endophytes for lignocellulolytic enzyme production and wood biodegradation. The mechanism for their growth as endophytes of roots and living standing trees is poorly understood, along with their initial colonization [154,155].

Basidiomycetous endophytes can take on a different life forms upon host death, as exemplified by *Schizophyllum commune* reported on oil palm [4,48], yet it is also a common saprophyte in the decay of various timbers [156,157] and a pathogen on apple trees (*Malus domestica*) [158] and grapevine trunks (*Vitis vinifera*) [159]. Robles et al. [160] studied the relationship between endophytic and pathogenic fungi which were originally isolated from wood samples and wood cores of *Platanus acerifolia*. There were two fungal genera including *Inonotus* spp. (Basidiomycota; three and six were endophytic and pathogenic strains, respectively) and *Daldinia* spp. (Ascomycota; three and two were endophytic and pathogenic strains, respectively), and their relationships were studied using three sets of experiments consisting of oxidase tests, in vitro wood-decaying tests, and phylogenetic analyses. All strains tested positive in the oxidase tests. Endophytic and pathogenic strains of *Inonotus* and *Daldinia* were inoculated onto dried wood blocks and incubated for three months, with all strains causing significant weight loss. All strains were phylogenetically related, but morphologically and phylogenetically indistinguishable. This suggests that ascomycetous and basidiomycetous endophytes can also switch their lifestyle between endophytes, saprophyte, and pathogens. 

Promputtha et al. [161] isolated fungi from leaves and twigs of *Magnolia liliifera* and grouped them as endophytes, sterile mycelia, and saprophytes, consisting of 41, 31, and 27 isolates, respectively. They found that four genera *Colletotrichum*, *Fusarium*, *Guignardia*, and *Phomopsis*, which are commonly found as endophytes in Ascomycota, were phylogenetically related to their saprophytic counterparts and had high sequence similarity. This provides a clue that some endophytes change their mode of living and adopt a saprophytic lifestyle after the death of host plants. Thus, some basidiomycetous endophytes may behave like ascomycetous endophytes, which supports the concept that endophytes live asymptomatically and mutualistically within host plants under normal circumstances but become pathogens or saprophytes upon host senescence.

### 5.5. Next-Generation Study

Metagenomics is an advanced combination method between molecular tools and computational software used in analyzing microbiomes from environmental samples without requiring axenic cultures [162]. Since then, various terms including known, unknown, identifiable, unidentifiable, culturable, and unculturable have been widely used to describe taxa found in genetic and diversity studies [163]. This expands the knowledge of the previously undetected microbes in environmental samples. Future diversity studies of endophytes will focus on metagenomic (high-throughput screening, HTS) methods for their detection and enumeration [163], and move away from traditional methods reliant on the isolation and sequencing of strains, which is time-consuming and often ineffective with slow-growing, fastidious, mycorrhizal fungi and unculturable fungi [10]. Metagenomics paves the way to discover more diverse groups of taxa, especially Basidiomycota and basal fungi [40,68]. This approach has already been successfully applied to studies of fungal communities in aquatic sediments, discovering novel chytrid and other fungal lineages [128,164,165,166,167]. 

Culture-dependent methods (CD method) and next-generation studies (NGS) have their own advantages and disadvantages. CD methods provide axenic cultures which can be further used in other industrial applications, and only culturable fungi can be recovered using this method. However, fastidious and unculturable ones are frequently omitted and this leads to a lower diversity of endophytes. Meanwhile, NGS offers insight and greater diversity of both culturable and unculturable mycoflora within the plant and environmental samples. But most are unculturable and unidentifiable, which makes this impossible for further application due to the lack of pure cultures for cross-referencing. Therefore, both methods are complementary, and if possible, both methods should be used for studies to illustrate the complete picture of endophyte diversity. 

## 6. Concluding Remarks

This review highlights how little is known about endophytic Basidiomycota in comparison to their Ascomycota counterparts. Much research of endophytes has been powered by bioprospecting studies for antimicrobials, with 59.6% of papers on the endophytes of the tree *Taxus* devoted to the production of taxol [2]. Such studies of endophytic Basidiomycota are few, and consequently, their role in the health of trees, shrubs, seaweeds, and seagrasses remains to be explored. For example, various endophytic basidiomycetes have been shown to produce antimicrobials: *Grammothele lineata*, from *Corchorus olitorius* (jute), produces paclitaxel with antifungal and antibacterial activities [168]; *Perenniporia tephropora* from *Taxus* sp. produces a cytotoxic albicanol [2]; and *Bjerkandera adusta* caused a 10.2% of mass loss in spruce wood chips after 8 weeks [152]. This clearly shows the potential of Basidiomycota to produce interesting compounds and is reason for greater effort to document their activity as endophytes.

Many reasons may account for this paucity of knowledge of endophytic Basidiomycota: 1. sterile isolates are not carefully examined; 2. selective media for their isolation are not used; 3. the incubation time is critical and needs to be as long as possible; 4. a wider range of host plants need to be studied; 5. culture-based studies do not always detect their presence; and 6. host tissues play an important role in the diversity of endophytes. Basidiomycetous endophytes are more likely to be found from woody substrates rather than leaves. 

Ascomycota are dominant in endophytic studies because of their potential as a source of antimicrobials (at least the asexual morphs) but Basidiomycota are equally important when it comes to the decay of wood [45]. The dominance of Ascomycota as endophytes can be attributed to their greater numbers, ease in dispersal, especially wind dispersal of asexual morphs, their ability to colonize a wider range of substrates, and their tolerance of extreme environments. Metagenomic-based studies reveal a much wider range of endophytic Basidiomycota and basal fungi, and thus, this will open a whole new area for future studies. What role do they play in health plant communities? Are they a source of enzymes and antimicrobials that protect host plants? How do they interact within their hosts in competition with Ascomycota? Are they hidden saprophytes waiting to colonize their senescent host? In the opening section of the paper, we queried the paucity of endophytic Basidiomycota, but clearly, this is not the case, as it very much depends on the plant tissue part under investigation. Foliar plant parts are dominated by Ascomycota, while Basidiomycota are found in branches, twigs, and woody tissues by culture-dependent methods, while geonomics methods reveal an even greater fungal diversity and require wider consideration and application. 

## Data Availability

The raw data supporting the conclusion of this article will be made available by the authors on request.

## Supplementary Materials

The following supporting information can be downloaded at: https://www.mdpi.com/article/10.3390/jof10010067/s1, Table S1: Species frequency of Basidiomycota and basal fungi as endophytes from selected 25 publications.
